# Sparse Constrained Reconstruction for Accelerating Parallel Imaging Based on Variable Splitting Method

**DOI:** 10.1155/2013/605632

**Published:** 2013-03-31

**Authors:** Wenlong Xu, Xiaofang Liu, Xia Li

**Affiliations:** Department of Biomedical Engineering, China Jiliang University, Hangzhou 310018, China

## Abstract

Parallel imaging is a rapid magnetic resonance imaging technique. For the ill-conditioned problem, noise and aliasing artifacts are amplified during the reconstruction process and are serious especially for high accelerating imaging. In this paper, a sparse constrained reconstruction problem is proposed for parallel imaging, and an effective solution based on the variable splitting method is contrived. First-order and second-order norm optimization problems are first split, and then they are transferred to unconstrained minimization problem by the augmented Lagrangian method. At last, first-order norm and second-order norm optimization problems are alternatively resolved by different methods. With a discrepancy principle as the stopping criterion, analysis of simulated and actual parallel magnetic resonance image reconstruction is presented and discussed. Compared with the routine parallel imaging reconstruction methods, the results show that the noise and aliasing artifacts in the reconstructed image are evidently reduced at large acceleration factors.

## 1. Introduction

Parallel imaging is a robust method for accelerating the acquisition of magnetic resonance imaging (MRI) data, which exploits spatial sensitivity of an array of receiver coils to reduce the number of the required Fourier encoding steps. However, these reduced amounts of MR data lead to aliased images by the routine reconstruction method. Over the past few years, a number of parallel MRI techniques have been proposed for reconstructing MR image from these undersampled data in either *k*-space or image domain [1]. Sensitivity encoding (SENSE) [2] and generalized auto-calibrating partially parallel acquisitions (GRAPPA) [3] are two methods most commonly used on clinical scanners today.

As the amount of data acquired in parallel MRI is less, which depends on the acceleration factor (AF), the quality of the reconstructed image is poorer. Therefore, the AF is usually lower when parallel imaging technique is used to speed up MRI in clinic. Based on SENSE method, the image reconstruction for parallel imaging is performed by solving a linear system that explicitly depends on the sensitivity maps of the receiver coils, and some prior information may be used to stabilize the reconstruction process. Regularization is an attractive means of restoring stability in the reconstruction mechanism, where prior information can be effectively incorporated [4]. The Tikhonov regularization is a commonly used method where a low-resolution prior image is applied in the reconstruction; that is, a quadratic minimization problem and its numerical algorithm are simple, such as the linear conjugate gradient (CG) method. However, the disadvantage of Tikhonov regularization method is that it biases the estimated reconstructed image towards the prior image [5]. More recently, total variation methods have been investigated for MR image reconstruction. The advantage of this type of regularization is that it biases the reconstructed image towards a piecewise smooth image, instead of a globally smooth image, thereby better preserving image edges [6]. With the advent of compressed sensing (CS) theory, sparsity-promoting regularization criteria have gained popularity in MRI, which is known as sparseMRI or CS-MRI [7]. The basic assumption underlying CS-MRI is that many MR images are inherently sparse in some transform domain and then can be reconstructed with high accuracy from significantly undersampled *k*-space data. Certainly, the CS framework is apt for pMRI with undersampled MR data [8].

This paper investigates the problem of sparse constrained reconstruction from highly undersampled MR data for parallel MRI. Based on sparseMRI theory, we use the finite difference as the sparsity project domain, and sparsity property of anisotropic total variation (TV) of MR image is used as the prior information for stabilizing the reconstruction process. As a result, a nonlinear optimization problem is constructed for reconstructing the parallel MR image. However, as there exists both first-order norm (known as *ℓ*1-norm) and second-order norm (*ℓ*2-norm) minimization problems, the solution to the constructed optimization problem is commonly difficult. In the paper, the *ℓ*1-norm minimization problem for sparse representation of MR image and *ℓ*2-norm minimization problem, which are subject to data consistency based on SENSE method, are firstly split by variable splitting method, and then the split constrained minimization problem is converted to an unconstrained minimization problem by the augmented Lagrangian (AL) method. At last, the Lagrangian multiplier method and alternating direction method (ADM) are used to solve the split minimization problem by the different numerical algorithms. In order to evaluate the effectiveness and robustness of the proposed algorithm, the image reconstruction problem from highly undersampled parallel MR data is exploited.

## 2. Theory

### 2.1. The MR Signal Model in Parallel Imaging

As a fast imaging method, parallel imaging technique is also known as multiple coils MRI, which uses an array of RF receiver surface coils to acquire multiple sets of undersampled *k*-space data simultaneously. Let $\overset{⃑}{r}$ denote the two-dimension spatial coordinates (*x*, *y*) and $s_{l}(\overset{⃑}{r})$ the demodulation information associated with the *l*th coil, then the MR signal associated with the *l*th coil is the following general forward model:

(1) $$\begin{array}{c} c_{l}\left(\overset{⃑}{r}\right)=\int s_{l}\left(\overset{⃑}{r}\right)f\left(\overset{⃑}{r}\right)e^{-i\overset{⃑}{k}\cdot \overset{⃑}{r}}d\overset{⃑}{r},\;l=1,\ldots ,L. \end{array}$$

Here, $f(\overset{⃑}{r})$ denotes the object's transverse magnetization signal and forms MR image. Let *y*-ordinates denote the gradient encoding direction, consideration of MR signal sampled on Cartesian coordinates, and then the discrete MR signal is obtained as follows (2) in accelerating parallel imaging:

(2) $$\begin{array}{c} I_{l}\left(x,y\right)=\sum_{n=0}^{N_{A}-1}s_{l}\left(x,y+nM\right)f\left(x,y+nM\right), \end{array}$$

where *N*_*A*_ is the number of aliased pixels; *R* is known as AF; *M* = *N*_*y*_/*R*; *x* = 0,…, *N*_*x*_; *y* = 0,…, *N*_*y*_/*R*; *N*_*x*_, *N*_*y*_ is the discrete pixels number, respectively, along the *x*-direction and *y*-direction when data are full sampled.

### 2.2. The Model of Image Reconstruction in Parallel Imaging

Considering of noisy samples of MR signal, the discrete model for parallel imaging is given as

(3) $$\begin{array}{c} y=FSf+\varepsilon , \end{array}$$

where *f* is a *N* × 1 column vector containing the samples of the unknown image to be reconstructed, *y* and *ε* are *ML* × 1 column vectors corresponding to the data samples from *L* coils and noise, respectively. *S* is *NL* × *N* matrix given by *S* = [*s*_1_^*H*^,…, *s*_*L*_^*H*^], *s*_*l*_ is *N* × *N* diagonal matrix corresponding to the sensitivity map of the *l*th coil, and 1 ≤ *l* ≤ *L*, (·)^*H*^ represents the Hermitian-transpose. *F* is *ML* × *NL* matrix given by *F* = *I*_*L*_ ⊗ Fu, Fu is *M* × *N* Fourier encoding matrix, *I*_*L*_ is the identity matrix of size *L*, and *⊗* denotes the Kronecker product. In order to speed up parallel imaging, *k*-space MR data may be undersampled to reduce the total scan time, so *M* ≤ *N*.

Given an estimate of the sensitivity maps *S*, the image reconstruction problem for parallel imaging is to find *f* from data *y*. Based on SENSE method, the MR image may be reconstructed using the least-squares estimation as

(4) $$\begin{array}{c} \hat{f}={\left(S^{H}F^{H}\Psi^{-1}FS\right)}^{-1}F^{H}S^{H}\Psi^{-1}y, \end{array}$$

where Ψ denotes the *L* × *L* receiver noise matrix, it describes the levels and correlation of noise in the receiver channels. Because of the coil configurations and the coil sensitivity error, the measurement matrix *FS* is commonly non-orthogonal as *S* ^−1^ *F* ^−1^ *FS* ≠ *I*. The linear system as (3) is ill-posed. Using inversion matrix method as (4), noise derived from measurement data may be amplified if the small eigenvalues exist in matrix *FS*, which might result in the instability of reconstruction process. As noise in MRI measurements is Gaussian distribution, a natural approach is to estimate *f* by minimizing a regularized least-squares cost function:

(5) $$\begin{array}{c} \hat{f}=\arg\;\min\left\{\Phi \left(f\right)={||FSf-y||}_{2}+\mu R\left(f\right)\right\}, \end{array}$$

where *R*(*f*) denotes the regularization term, and ||||_2_ is *ℓ*2-norm, which represents the data-consistent term. *μ* is the so-called regularization parameter to balance the regularization term and data-fidelity term.

An open problem in most regularization image reconstructions is how to best choose the regularization term. If this term is not included, then the image estimate will suffer from noise and aliasing artifacts for undersampled data. The simplest choice is the Tikhonov regularization *R*(*f*) = ||*f* | |_2_ or *R*(*f*) = ||*f* − *f*_0_ | |_2_, where *f*_0_ is a prior or reference image. However, if the reference image is zero, then all pixel values in estimation *f* are diminished towards zero, possibly reducing contrast. Another choice is a quadratic roughness penalty function, by which it is convenient for minimization and guarantees that the cost function (5) has a unique minimization, but it has the drawback of smoothing image edges. TV regularization reconstruction may overcome the drawbacks mentioned above, but it is getting harder to be minimized and can lead to the appearance of “blocky” texture in images.

### 2.3. The Sparse Constrained Image Reconstruction in Parallel Imaging

Based on CS-MRI theory, MR image on finite difference domain is sparse, on which the sparse representation of MR images can be demonstrated by applying a sparsifying transform to a fully sampled image. In this paper, TV transform is used to transform the estimated MR image to finite difference domain, and then the sparse constrained minimization problem for parallel MR image reconstruction is obtainedas

(6) $$\begin{array}{c} \underset{f}{\min}{||\nabla f||}_{1}\;\text{s.t.}\;\;{||FSf-y||}_{2}<\sigma , \end{array}$$

where ||||_1_ denotes *ℓ*1-norm, and *σ* represents any error, such as noisy level derived from the data sampled process. ||∇*f* | |_1_ is called TV norm of an image, which is defined as a function of the image gradient. ||∇*f* | |_1_ might be computed by discrete isotropic TV or anisotropic TV as

(7) $$\begin{array}{c} {||\nabla f||}_{1}=\sum_{i}\sqrt{{\left(\nabla_{x}f\right)}_{i}^{2}+{\left(\nabla_{y}f\right)}_{i}^{2}},\\ {||\nabla f||}_{1}=\sum_{i}\left(\left|\nabla_{x}f\right|+\left|\nabla_{y}f\right|\right), \end{array}$$

where ∇_*x*_ *f* denotes the *x*-direction gradient of image *f*, and ∇_*y*_ *f* denotes the *y*-direction gradient. There are both *ℓ*1-norm term and *ℓ*2-norm term in (6). The *ℓ*1-norm term in (6) is based on the sparsity of MR image on finite difference domain, while *ℓ*2-norm term is based on the data fidelity of MR image reconstruction. Using the common numerical algorithms such as CG or Newton method, it is difficult to attain the stabilized solution to problem (6). Lustig et al. researched sparseMRI and posed the detailed computation method for sparse constrained inverse problem [8], where nonlinear CG method was applied to solve the constrained minimization problem as (6) when isotropic TV is applied to compute TV. However, the reconstructed image obtained by this method is dissatisfied according to our analysis.

According to the research product about *ℓ*1-norm minimization problem [9–11], the variable split method is adopted to split the *ℓ*1-norm term and *ℓ*2-norm term in (6) in our research. Specifically, with an auxiliary variable *w*, let *w* = ∇*f*, then the constrained optimization problem is obtained, which denoted as *P* as follows:

(8) P:min⁡w,f||w||1 s.t.  w=∇f,  ||FSf−y||2<σ.

## 3. The Solution to Sparse Constrained Image Reconstruction Based on Variable Splitting Method for Parallel Imaging

### 3.1. The Unconstrained Parallel MR Image Reconstruction Problem

In the augmented Lagrangian (AL) framework (also known as the multiplier method [12]), an AL function can be constructed for problem (8) as

(9) min⁡w,f,γ1,γ2Φ(f,w,γ1,γ2)=||w||1+γ1H(FSf−y)+μ12||FSf−y||2+γ2H(w−∇f)+μ22||w−∇f||2,

where *γ*_1_, *γ*_2_ represent the vector of Lagrange multipliers, and *μ*_1_, *μ*_2_ are the regularization parameters. The solution to (9) may be in the following AL version:

(10) $$\begin{array}{c} \left(f^{k+1},w^{k+1}\right)⟵\underset{f,w}{\arg\;\min}\;\Phi \left(f,w,\gamma_{1}^{k},\gamma_{2}^{k}\right),\\ \gamma_{1}^{k+1}⟵\gamma_{1}^{k}-\lambda \mu_{1}\left(FSf^{k+1}-y\right),\\ \gamma_{2}^{k+1}⟵\gamma_{2}^{k}-\lambda \mu_{2}\left(w^{k+1}-\nabla f^{k+1}\right), \end{array}$$

where *λ* ∈ (0,2) guarantees convergence, as long as the subproblem is solved to an increasingly high accuracy at every iteration.

The joint minimization of Φ with respect to *f* and *w* can be computationally challenging in (10). ADM [13] is applied, which alternatively minimizes Φ with respect to one variable at a time while holding others constant. This method decouples the individual updates of *f* and *w* and simplifies the optimization task. Specifically, at the *k*th iteration, we perform the following individual minimizations, taking care of using updated variables for subsequent minimizations and the following algorithm:

(11) $$\begin{array}{c} w^{k+1}⟵\underset{w}{\arg\;\min}\;\Phi \left(f^{k},w,\gamma_{2}^{k},\mu_{2}\right),\\ f^{k+1}⟵\underset{f}{\arg\;\min}\;\Phi \left(f,w^{k+1},\gamma_{1}^{k},\mu_{1},\gamma_{2}^{k},\mu_{2}\right),\\ \gamma_{1}^{k+1}⟵\gamma_{1}^{k}-\lambda \mu_{1}\left(FSf^{k+1}-y\right),\\ \gamma_{2}^{k+1}⟵\gamma_{2}^{k}-\lambda \mu_{2}\left(w^{k+1}-\nabla f^{k+1}\right). \end{array}$$

### 3.2. The Solution to Minimization Problem with Respect to *w*

Holding variable *f* constant, we get the minimization problem with respect to *w* in (9) at the *k*th iteration as

(12) wk+1=arg min⁡w{||w||1+(γ2k)H(w−∇fk)+μ22||w−∇fk||2}.

Equation (12) is a *ℓ*1-norm minimization problem, the solution of which can be estimated by shrinkage rule [14] as

(13) wk+1=Shrink{∇fk−γ2kμ2,1μ1∗μ2}≜max⁡{|∇fk−γ2kμ2|−1μ1∗μ2,0}·sgn⁡(∇fk−γ2kμ2).

### 3.3. The Solution to Minimization Problem with Respect to *f*

Holding variable *w* constant, we get the minimization problem with respect to*f* at the *k*th iteration based on (9) as

(14) fk+1=arg min⁡f{(γ1k)H(FSf−y)+μ12||FSf−y||2      +(γ2k)H(wk−∇f)+μ22||wk−∇f||2}.

Ignorant of irrelevant constant, (14) can be written as (15)

(15) $$\begin{array}{c} f^{k+1}=\underset{f}{\arg\;\min}\left\{\frac{\mu_{1}}{2}{||FSf-y-\eta_{1}||}_{2}+\frac{\mu_{2}}{2}{||w^{k}-\nabla f-\eta_{2}||}_{2}\right\}, \end{array}$$

where *η*_1_ = *γ*_1_/*μ*_1_, *η*_2_ = *γ*_2_/*μ*_2_. Equation (15) is a *ℓ*2-norm minimization problem, which can be solved by nonlinear conjugate gradient (NCG) descent algorithm with backtracking line search algorithm. During the iterative process, the iterative step may be calculated by inexact line search or the Barzilai and Borwein (BB) method [15].

### 3.4. The Solution to Sparse Constrained Image Reconstruction for Parallel Imaging

Combining the results in Sections 3.1, 3.2, and 3.3, we now present the algorithm for solving the unconstrained optimization problem (9) as follows.

(1) Initialize *f* ^0^, *w* ^0^ and regularization parameters *μ*_1_, *μ*_2_.
(2) Precompute *F* ^*H*^ *S* ^*H*^ *f*, and let *η*_1_^0^ = *η*_2_^0^ = 0, *k* = 0.
  Repeat the following.
(3) Obtain an update *w* ^*k*+1^ using an appropriate technique as described in Section 3.2.
(4) Update *f* ^*k*+1^ using an appropriate technique as described in Section 3.3 by NCG method, and the inexact line research method is used to calculate the iterative step.
(5) Update the Lagrangian multiplier *γ*_1_ by the following function:
  (16) $$\begin{array}{c} \gamma_{1}^{k+1}⟵\gamma_{1}^{k}-\lambda \mu_{1}\left(FSf^{k+1}-y\right). \end{array}$$
(6) Update the Lagrangian multiplier *γ*_2_ by the following function:
  (17) $$\begin{array}{c} \gamma_{2}^{k+1}⟵\gamma_{2}^{k}-\lambda \mu_{2}\left(w^{k+1}-\nabla f^{k+1}\right). \end{array}$$
(7) Let *k* = *k* + 1.

Until some stop criterion is met.

## 4. Experiments

### 4.1. Data Simulation and Acquisition

In all our experiments, we considers *k*-space MR data assigned on the Cartesian grid, and the undersampling pattern is uniformity, so Fourier encoding matrix corresponds to an undersampling version of the DFT matrix. Two sets of parallel MR data are applied to test the proposed algorithm. One set of data is acquired from simulating parallel MRI system, and the other is actual parallel MR data.

In order to construct the simulating multichannel MRI receive system, we considered a noise-free 256 × 256 T1-weighted MR image obtained from the BrainWeb database [16] and then downsampled to 128 × 128. The simulating system had four-channel linear phased-array coil wrapped around the whole brain circumferentially. The coil sensitivity maps were calculated using Biot-Savart's law [17]. Multichannel images were then created by multiplying T1-weighted MR image with the simulated sensitivity profiles. At last, these images were Fourier transformed to generate the multichannel *k*-space data whose size was 128 × 128 × 4. In the research, complex Gaussian noise would be added to data set for simulating noisy parallel MR data.

The actual fully sampled brain dataset was obtained from PULSAR (a Matlab toolbox for parallel MRI) [18], which was acquired using MR systems with eight-channel head array and multichannel receiver from a healthy male volunteer with fast spoiled gradient-echo sequence, TR/TE = 300/10 ms, matrix size = 256 × 256, tip angle = 15°, and FOV = 22 × 22 cm.

To simulate the accelerating imaging procedure for parallel MRI, *k*-space dataset along phased encoding direction might be uniformly decimated to produce undersampling datasets. Additionally, central 12 phase-encoding lines were preserved to be used for coil sensitivity estimation [19]. We mainly researched parallel MR image reconstruction problem when the undersampling rate (also known as AF) attained its maximum value that was the number of receive coils at theory. The size of undersampled data in simulated data analysis is 128 × 32 × 4, while actual dataset is 256 × 32 × 8. Meanwhile, for two sets of data, the full *k*-space data was acquired and then sum-of-squares (SoS) reconstruction [20] was used as a reference image.

**Figure 1:**
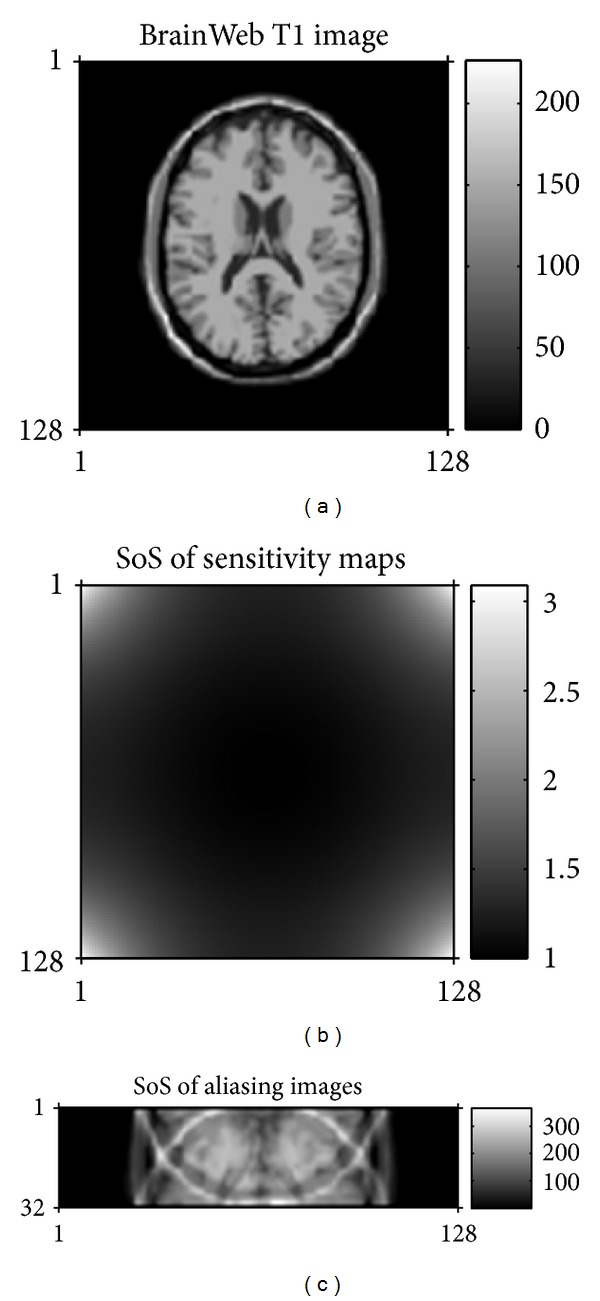
Simulated pMRI. (a) is the T1-weighted brain MR image, (b) is SoS of individual receiver coil sensitivity profiles calculated based on Biot-Savart's law, and (c) is SoS reconstruction from undersampled data when AF is 4.

Figure 1(a) shows the data collected from the simulating parallel MRI, which will be applied to test the proposed algorithm.

### 4.2. Calibration of Sensitivity Profiles

Calibration of the spatial sensitivity functions of coil arrays is a crucial element in parallel MRI. Accurate coil sensitivity information is required for accurate spatial encoding in parallel MRI reconstructions, and the choice of sensitivity calibration strategy is at least as important as the choice of reconstruction strategy. The most common approach has been to measure coil sensitivities directly using one or more low-resolution images acquired before or after accelerated data acquisition. However, since it is difficult to ensure that the patient and coil array will be in exactly the same positions during both calibration scans and accelerated imaging, this approach can introduce sensitivity miscalibration errors into parallel MRI reconstructions. Coil sensitivity functions vary slowly as a function of spatial position, and low-resolution in vivo images suffice to form sensitivity references. Thus, valid coil sensitivities can be determined from the fully sampled region of the central *k*-space, so long as the range of spatial frequencies covered in the central region contains the spatial frequency band of the coil sensitivity functions [19]. In the paper, central 12 phase-encoding lines were Fourier transformed to produce low-resolution $f(\vec{r})$, which was used to estimate the coil sensitivity profiles. By (18), the magnetization distribution low-resolution $f(\overset{⃑}{r})$ may be partly removed, and then the encoding effects of relatively pure coil sensitivities are isolated as

(18) $$\begin{array}{c} {\hat{s}}_{\text{l}}\left(\overset{⃑}{r}\right)\approx \frac{{\left[f\left(\overset{⃑}{r}\right)s_{\text{l}}\left(\overset{⃑}{r}\right)\right]}^{\text{low-resolution}}}{\sqrt{\sum_{l}{\left|{\left[f\left(\overset{⃑}{r}\right)s_{\text{l}}\left(\overset{⃑}{r}\right)\right]}^{\text{low-resolution}}\right|}^{2}}}, \end{array}$$

where the “low-resolution” superscript indicates that use of only the central *k*-space positions results in a low-resolution measurement of the full product of $f(\overset{⃑}{r})$ and $s_{l}(\overset{⃑}{r})$.

### 4.3. Data Analysis

In the study of actual parallel MRI, the standard reference image is the SoS reconstructed image of full-sampled individual coil surface images, while the T1-weighted brain image is the reference image in the simulating study. The normalized mean squared error (NMSE) between the reconstructed image and the reference image would be calculated to quantitatively analyze the quality of reconstructed image. NMSE is also known as artifact power (AP), which suggests any error as both increased image artifacts and noise. As shown in (19), a higher value of NMSE (or AP) represents the reduced image quality as

(19) $$\begin{array}{c} \text{NMSE}=\frac{\sum_{\overset{⃑}{r}}{\left(\left|f\left(\overset{⃑}{r}\right)\right|-\left|f^{\text{reference}}\left(\overset{⃑}{r}\right)\right|\right)}^{2}}{{\sum_{\vec{r}}{\left|f^{\text{reference}}\left(\overset{⃑}{r}\right)\right|}^{2}}^{}}, \end{array}$$

where *f* ^reference^ denotes the sensitivity reference image.

To pose the effectiveness of the proposed reconstruction method as (9), the other parallel MR image reconstruction methods known as generalized encoding matrix (GEM) method [21], which is used in SENSE method, and CG method [22] are comparatively analyzed. Additionally, the stopping criterion for the iterative algorithms used in our study is based on discrepancy principle as

(20) $$\begin{array}{c} \frac{\mathrm{norm}\left(f_{j}-f_{j-1}\right)}{\mathrm{norm}\left(f_{j}\right)}<\text{tol}, \end{array}$$

where description “norm” indicates Frobenius norm of matrix, “tol” denotes the convergence tolerance, and subscription “*j*” indicates the iteration number during the iterative process.

### 4.4. Results

**Figure 2:**
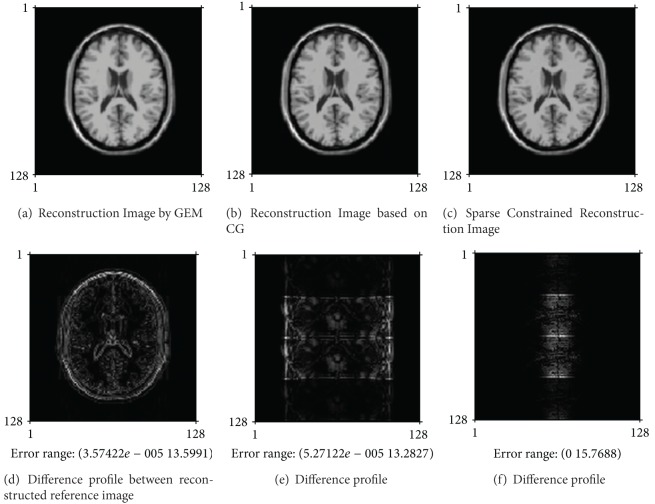
Reconstructed images from noise-free undersampling MR data for the simulated parallel MRI, where the undersampling rate is 4, which is the maximum of AF at theory. The top three images are, respectively, reconstructed by GEM method, CG method, and the proposed method as (9), which is solved by the algorithm referred to in Section 3.4. The bottom three images are the difference profiles between the above three reconstructed images and the reference image.

**Figure 3:**
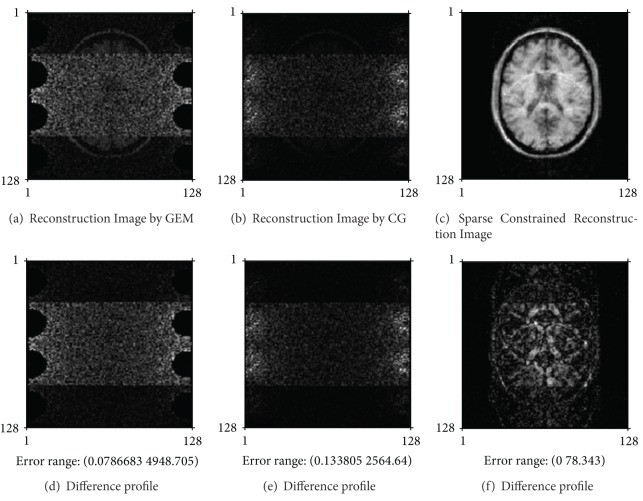
Reconstructed images from noisy undersampled MR data for the simulated parallel MRI, where the undersampling rate (named AF) is 4.

We compared the proposed method to GEM method, named the basic SENSE, and to CG method, by which MR images may be reconstructed from undersampled MR data.  Figure 2 shows the reconstructed images from noise-free simulating data when employing 4-fold accelerating MRI, when AF is 4.  Figure 3 shows the reconstructed images from noisy 4-fold undersampled MR data, where the added noise is zero-mean Gaussian noise. In Figures 2 and 3, the three images on the top are the reconstructed images by the methods as mentioned above, and the images on the bottom are the difference profiles between the top three reconstructed images and reference image.

During the iterative process of reconstructing the images as seen in Figures 2 and 3, we let the convergence tolerance 0.0001. When the proposed algorithm (described in Section 3.4) is used to reconstruct the image in Figure 2, the penalty parameters *μ*_1_, *μ*_2_ are 2^−27^, 2^−33^, respectively. The number of iterations is 20 for resolving the minimization problem with respect to *f* by the NCG method referred to in Section 3.3, and the total number of iterations for the proposed algorithm is 411, namely, the valuable *k* referred to in Section 3.4. At the same time, the proposed algorithm is used to reconstruct the images in Figure 3, and the penalty parameters *μ*_1_, *μ*_2_ are 2^−26^, 2^−22^, respectively, and the number of iteration is 20 for resolving the minimization problem with respect to *f* by NCG method, and the total number of iterations for the algorithm proposed is 198. Tables 1 and 2 are the mean of NMSE, respectively, in Figures 2 and 3.

As shown in Figure 2, the proposed algorithm may obviously restrain the aliasing artifacts resulted from undersampled MR data. As seen in Figure 3, noise derived from measurement procedure is suppressed and result in the convergence of iterative process by our proposed algorithm.

**Figure 4:**
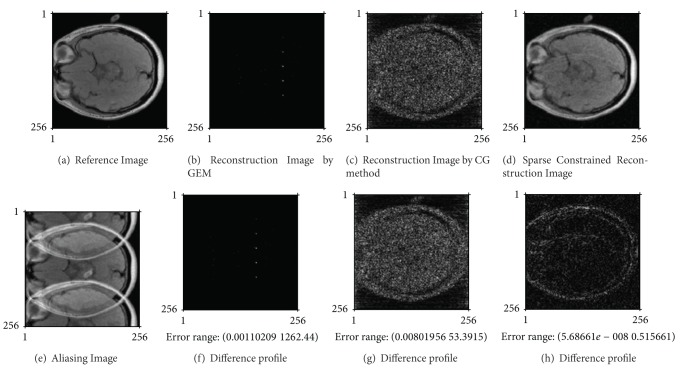
Reconstructed images from noisy undersampling MR data for in vivo parallel MRI, where the undersampling rate is 8, which is the maximum at theory. (a) is the reference image (SoS reconstruction of full-sampled coil images). (b), (c), and (d) are images reconstructed, respectively, by GEM method, CG method, and the proposed method proposed as (9). (e) is the reconstructed image by SoS method. (f), (g), and (h) are the difference profiles, respectively, between (b), (c), (d), and (a).

In analysis of actual parallel MR image reconstruction, AF is set as 8, which is the maximum at theory for parallel MRI system with 8-channel coil array.  Figure 4 shows the reconstructed image and the difference profiles between reconstructed images and the reference image, and the mean of NMSE is shown in Table 3.

In Figure 4, the left image on the top penal is the reference image, and the left image on the bottom is the reconstructed image by SoS method from 8-fold undersampled parallel MR data. During iterative process of reconstructing the images in Figure 4, the convergence tolerance is 0.001. When the proposed algorithm is used to reconstruct the image in Figure 4, the penalty parameters *μ*_1_, *μ*_2_ are 2^−20^, 2^−11^, respectively. The iterative number is 1 for resolving the minimization problem with respect to *f* by NCG method, and the total iterative number of algorithm proposed is 37.

As shown in Figures 3 and 4, the proposed algorithm can effectively reduce the aliasing artifacts and noise information.

## 5. Discussion

The quality of the reconstructed image for parallel imaging may be affected by the undersampling rate, noise level, the exact estimation of receiver coils sensitivity, *k*-space data trajectory, and reconstruction method. We only researched the image reconstruction from undersampled *k*-space data acquired along the Cartesian trajectory. However, the proposed algorithm is also fit to reconstruct MR image for arbitrary trajectories. 

There may be two approaches to improve the quality of the reconstructed image based on SENSE method: one approach is to improve the accuracy estimation of sensitivity, another approach is to propose the effective reconstruction algorithm. However, when AF is large, only by improving the reliability of the coil sensitivity maps, the suppression of aliasing artifact and noise in the reconstructed image is indistinctive. In order to remarkably improve the quality of the reconstructed image, the constrained reconstruction is generally shown to be an effective method. The goal of our proposed method is to improve the quality of the reconstructed image, when MR data are highly undersampled in parallel imaging. As the coil sensitivity profiles are already obtained, the reconstruction problem in parallel imaging is formulated as solving a set of linear equations based on (3). These equations can be very ill conditioned depending on the coil configurations and sampling trajectories, which further deteriorate the quality of the reconstructed image, especially when high undersampling rate is used for accelerating parallel imaging procedure. Therefore, the ill-conditioned problem in parallel imaging has been partially addressed by optimizing coil geometry, optimizing sampling trajectory, or introducing regularizations. In this paper, the ill-conditioned problem is partly reformed by adding the constrained condition to stabilize the reconstruction process. As anywhere noise exits, the reconstruction method for parallel imaging should be proposed on full consideration of both noise and aliasing artifacts suppressed problem. Based on sparseMRI theory, as MR image is sparse on finite difference domain, and a sparse constrained reconstruction problem for parallel imaging is constructed in the paper. Through the methods proposed and the experiment presented, the following aspects could be taken into account.

(1) Compare the proposed reconstruction method with the other methods for parallel imaging. According to our analysis results, the GEM algorithm for usual SENSE method is usually suitable for noise-free data and the accelerating imaging procedure at low AF. CG method can effectively restrain aliasing artifacts in the reconstructed image and be fast convergence on condition of noise-free undersampled data. However, when MR image may be reconstructed from the noisy and undersampled MR data, NMSE value between the reconstructed image and reference image first rapidly decreases and then gradually increases along with CG iterative reconstruction process. As shown in Figure 3, the least mean of NMSE between the reconstructed image by CG method and the reference image was 0.0762 after 21 numbers of CG iteration, but reached the convergence condition after 975 numbers of iteration. Similarly in Figure 4, the least mean of NMSE of the reconstructed image by CG method was 0.0623, but reached the convergence condition after 1020 number of iteration, and the least mean of NMSE was shown in Table 3.

(2) Reconstruct MR image under the nonnegative constraint condition. In our proposed algorithm, the solution to subproblem with respect to *f* as (15) is a quadratic minimization problem, which may be the following subproblem on condition of nonnegative constraint:

(21) min⁡f{μ12||FSf−y−η1||2+μ22||wk−∇f−η2||2}s.t.  real(f)>0,

where real ( ) indicators the operator of getting the real of complex *f*.

**Figure 5:**
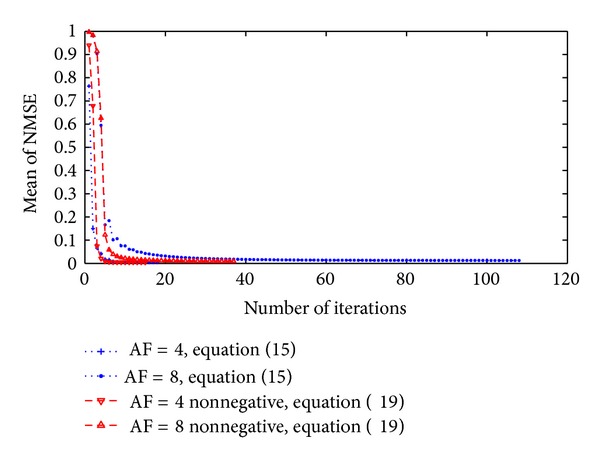
Iteration convergence process of reconstructing MR image for actual parallel MRI. The reconstruction method is the proposed algorithm described in Section 3.4, and (15) and (21) are, respectively, used for the subproblem with respect to *f*, where NCG descent algorithm with backtracking line search algorithm is used to solve (15) and (21).

Figure 5 shows the iteration process during reconstructing MR image *f* in analysis of actual parallel MRI, where (15) and (21) are, respectively, used in the proposed algorithm described in Section 3.4. As shown in Figure 5, when reconstructing image under the nonnegative constraint condition, the iteration process described in Section 3.4 might rapidly converge, and the mean of NMSE was reduced between the reconstructed image and the reference image.

(3) Reconstruct MR image at the different AFs for parallel imaging. Parallel MRI utilizes a radio frequency (RF) coil array to simultaneously acquire data from multiple receivers, and acceleration is achieved by a reduced phase encoding *k*-space trajectory. The nature of the subsampled *k*-space data requires the use of a reconstruction algorithm to restore aliased images into full field-of-view (FOV) images.  Table 4 is the mean NMSE of reconstructed image, respectively, by GEM method, CG method and the proposed algorithm at the different AFs.

As shown in Table 4, the proposed algorithm obviously improves the quality of reconstructed images. Compared to Table 3, AF is the main reason for affecting the quality of reconstructed image, so aliasing artifacts suppression may be chiefly considered for reconstruction method.

(4) The solution to the quadratic minimization problem is as follows. The solution to subproblem with respect to *f* as (15) is a quadratic minimization problem, which can be solved by the steepest descent method, the Newton method, or CG method. However, when resolving the subproblem with respect to *f*, theNewton method is inferior to the nonlinear CG method by the data analysis results.

(5) Penalty parameters and iteration convergence problem for regularization reconstruction are as follows. Convergence problem should be firstly considered for any iteration algorithm. The proposed algorithm for reconstructing MR image is an iterative solution course, and penalty parameters *μ*_1_, *μ*_2_ greatly affect the iterative convergence process. As long as penalty parameters chosen are suitable, the proposed algorithm can rapidly converge. Thereby, the penalty parameters *μ*_1_, *μ*_2_ are the key factors of optimization problem of sparse constrained reconstruction as shown in (9), which is a *ℓ*1-norm and *ℓ*2-norm minimization problem, named *ℓ*1-*ℓ*2 optimization problem. The common L-curve method is unable to determine the penalty parameters *μ*_1_, *μ*_2_. According to the quantitative index as NMSE between the reconstructed image and the reference image, we explored the better suitable penalty parameters within a determinate range. Specifically, the ratio of *μ*_1_ to *μ*_2_ was fixed, and *μ*_1_, *μ*_2_ increased by power of 2 in the range of 2^−30^ to 2^10^. The penalty parameters *μ*_1_, *μ*_2_ would be selected in our research, when they were used to (9) and then the least mean of NMSE between the reconstructed image and the reference image can be obtained.

## 6. Conclusion

In order to improve the quality of the reconstructed image for parallel imaging, a sparse constrained image reconstruction algorithm based on variable splitting method is proposed. Through the analysis of reconstructing full-FOV image from simulating and actual parallel MR data, the proposed algorithm can effectively suppress the aliasing artifacts in the reconstructed image resulted from the undersampled MR data, and also noise is obviously suppressed and the edge of image is preserved.

## Figures and Tables

**Table 1:** Mean of NMSE of reconstruction image (in Figure 2).

Reconstruction method	GEM	CG	Sparse constrained reconstruction
Mean of NMSE	4.1985*e* − 004	2.8799*e* − 004	2.2580*e* − 004

**Table 2:** Mean of NMSE of reconstruction image (in Figure 3).

Reconstruction method	GEM	CG	Sparse constrained reconstruction
Mean of NMSE	2.8860	12.5720	0.0248

**Table 3:** Mean of NMSE of reconstructions image from actual pMRI data (AF = 8, in Figure 4).

Reconstruction methods	GEM	CG	Sparse constrained reconstruction
NMSE	356.7079	343.1923	0.0095

**Table 4:** The mean of NMSE of reconstructed image from actual pMRI data (AF = 4).

reconstruction	GEM	CG reconstruction	Sparse constrained reconstruction
NMSE (noise-free data)	0.7641	0.0024	0.0019
NMSE (noisy data)	0.6698	85.2921	0.0049

## References

[1] Deshmane A, Gulani V, Griswold MA, Seiberlich N (2012). Parallel MR imaging. *Journal of Magnetic Resonance Imaging*.

[2] Pruessmann KP, Weiger M, Scheidegger MB, Boesiger P (1999). SENSE: sensitivity encoding for fast MRI. *Magnetic Resonance in Medicine*.

[3] Griswold MA, Jakob PM, Heidemann RM (2002). Generalized autocalibrating partially parallel acquisitions (GRAPPA). *Magnetic Resonance in Medicine*.

[4] Omer H, Dickinson R (2011). Regularization in parallel MR image reconstruction. *Concepts in Magnetic Resonance A*.

[5] Lin FH, Wang FN, Ahlfors SP, Hämäläinen MS, Belliveau JW (2007). Parallel MRI reconstruction using variance partitioning regularization. *Magnetic Resonance in Medicine*.

[6] Block KT, Uecker M, Frahm J (2007). Undersampled radial MRI with multiple coils. Iterative image reconstruction using a total variation constraint. *Magnetic Resonance in Medicine*.

[7] Lustig M, Donoho D, Pauly JM (2007). Sparse MRI: The application of compressed sensing for rapid MR imaging. *Magnetic Resonance in Medicine*.

[8] http://www.stanford.edu/~mlustig/SparseMRI.html.

[9] Zhang X, Burger M, Bresson X, Osher S (2010). Bregmanized nonlocal regularization for deconvolution and sparse reconstruction. *SIAM Journal on Imaging Sciences*.

[10] Yin W, Osher S, Darbon J (2008). Bregman iterative algorithms for compressed sensing and related problems. *SIAM Journal on Imaging Sciences*.

[11] L1-Related Optimization Project. http://www.caam.rice.edu/~optimization/L1.

[12] Afonso MV, Bioucas-Dias JM, Figueiredo MAT (2010). Fast image recovery using variable splitting and constrained optimization. *IEEE Transactions on Image Processing*.

[13] Ramani S, Fessler JA (2011). Parallel MR image reconstruction using augmented lagrangian methods. *IEEE Transactions on Medical Imaging*.

[14] Hale ET, Yin W, Zhang Y (2008). Fixed-point continuation for 11-minimization: methodology and convergence. *SIAM Journal on Optimization*.

[15] Vogel CR (2002). *Computational Methods for Inverse Problems*.

[16] Brainweb Simulated MRI volumes for normal brain. http://www.bic.mni.mcgill.ca/brainweb/.

[17] Grivich MI, Jackson DP (2000). The magnetic field of current-carrying polygons: an application of vector field rotations. *The American Journal of Physics*.

[18] Ji JX, Jong BS, Rane SD (2007). PULSAR: a MATLAB toolbox for parallel magnetic resonance imaging using array coils and multiple channel receivers. *Concepts in Magnetic Resonance B*.

[19] McKenzie CA, Yeh EN, Ohliger MA, Price MD, Sodickson DK (2002). Self-calibrating parallel imaging with automatic coil sensitivity extraction. *Magnetic Resonance in Medicine*.

[20] Roemer PB, Edelstein WA, Hayes CE, Souza SP, Mueller OM (1990). The NMR phased array. *Magnetic Resonance in Medicine*.

[21] Pruessmann KP (2006). Encoding and reconstruction in parallel MRI. *Magnetic Resonance in Medicine*.

[22] Pruessmann KP, Weiger M, Börnert P, Boesiger P (2001). Advances in sensitivity encoding with arbitrary k-space trajectories. *Magnetic Resonance in Medicine*.

